# Endoscopic vacuum therapy

**DOI:** 10.1016/j.igie.2024.06.003

**Published:** 2024-09-13

**Authors:** the American Society for Gastrointestinal Endoscopy Technology Committee, Samuel Han, Mohit Girotra, Maaza Abdi, Venkata S. Akshintala, Dennis Chen, Yen-I Chen, Koushik K. Das, David J. Desilets, Daniela Guerrero Vinsard, Galen Leung, Girish Mishra, V. Raman Muthusamy, Frances U. Onyimba, Swati Pawa, Tarun Rustagi, Sonali Sakaria, Nikrad Shahnavaz, Ryan J. Law

**Affiliations:** 1 Department of Gastroenterology and Hepatology, Mayo Clinic, Rochester, Minnesota, USA; 2 Digestive Health Institute, Swedish Medical Center, Seattle, Washington, USA; 3 Digestive Health Institute, Swedish Medical Center, Seattle, Washington, USA; 4 Digestive Diseases Center, University of Chicago, Chicago, Illinois, USA; 5 Division of Gastroenterology & Hepatology, McGill University Health Centre, Montreal, Quebec, Canada; 6 Division of Gastroenterology, Washington University School of Medicine, St Louis, Missouri, USA; 7 Division of Gastroenterology, Baystate Medical Center, Springfield, Massachusetts, USA; 8 Department of Gastroenterology, University of Pennsylvania, Philadelphia, Pennsylvania, USA; 9 Department of Gastroenterology, Wake Forest School of Medicine, Winston Salem, North Carolina, USA; 10 Division of Digestive Diseases, UCLA Health System, David Geffen School of Medicine at UCLA, Los Angeles, California, USA; 11 Department of Gastroenterology, WellSpan Digestive Health, York, Pennsylvania, USA; 12 Department of Gastroenterology, California Pacific Medical Center, San Francisco, California, USA; 13 Division of Digestive Diseases, Emory University, Atlanta, Georgia, USA

Endoscopic vacuum therapy (EVT) is a rapidly evolving novel technology increasingly used in the GI tract for management of endoscopic and surgical adverse events such as perforations, leaks, and fistulae. Initially recognized by plastic surgeons as a management option for chronically infected and ischemic wounds, vacuum therapy has experienced rapid adoption into other specialties, including gastroenterology.^1^ In this American Society for Gastrointestinal Endoscopy technology document, we discuss the indications, technical aspects, and outcomes of EVT for the management of various GI conditions.

When used externally, vacuum therapy comprises the application of negative pressure to a secondary healing wound using a sealed system that includes a sponge applied to the wound, an airtight film covering the sponge, and a suction pump connected to the sponge by plastic tubing. This technique promotes wound healing and accelerates defect closure using various mechanisms including but not limited to increased blood flow; local modulation of cytokines and chemoreceptor-modulated cell signaling, resulting in improved neoangiogenesis and granulation tissue growth; removal of debris and microorganisms; reduction of interstitial edema; continuous drainage of wound secretions; and macro-deformation of the wound with approximation of its edges and reduction of its volume.^2^

When used for GI indications, the sponge is attached to drainage tubing and endoscopically maneuvered to the defect site. On the application of vacuum pressure, the cavity collapses around the sponge, thus sealing the system without the need for a covering film. This approach was first implemented in 2003 in Germany for the management of rectal wound dehiscence and an infected mesorectal cavity.^3^ In 2008, EVT was used in the upper GI tract, with subsequent expansion of uses and indications thereafter.^3^, ^4^, ^5^

EVT can be applied to any luminal defects in the GI system, provided there is a closed compartment to collapse around the negative pressure device and adequate vascular perfusion exists around the defect to allow tissue healing. The size of the defect should be considered when planning EVT because this may predict the duration of therapy. Available data support the successful use of EVT in cavities .3 cm to 15 cm in length.^6^^,^^7^ Once established, the EVT system is changed regularly until adequate healing is achieved and the defect closes.

## Indications

EVT is most commonly used for the management of leaks and perforations, more commonly in the upper GI tract compared with the lower GI tract. The common indications for EVT are anastomotic leak after upper GI surgery, leaks after bariatric surgery, duodenal defects, acute perforations (iatrogenic, spontaneous, or traumatic), colonic wall defects and fistulae, and prophylactic EVT after major surgery.^8^ The outcomes of EVT for these specific indications are discussed in more detail below.

## Contraindications

EVT is contraindicated in clinical scenarios where it will prove ineffective or pose great risk to the patient. This includes connection of the GI lumen to the airway or atmosphere where the vacuum will be unable to maintain a seal. Examples include a tracheoesophageal fistula where negative pressure would divert air from the airway or a fistula to a cavity that is connected to the skin. In both examples, EVT would not form an effective seal. Additionally, EVT should not be performed if a visible vessel is identified near the defect or in cases where major bleeding occurs during the course of performing EVT.

## Technique for EVT

Although there are a variety of methods to perform EVT, the basic technique entails attaching a sponge to a nasogastric tube (NGT), which can then be endoscopically placed at the site of the luminal defect. Continuous suction is then maintained with the sponge replaced every 3 to 5 days until healing of the defect is achieved. EVT performed in the upper GI tract is typically performed with the patient under general anesthesia with a secure airway, whereas EVT in the lower GI tract can be performed with the patient under deep sedation. Although patients typically receive antibiotics throughout the duration of treatment for GI leaks and perforations, intraprocedural antibiotics during EVT are not routinely administered.^9^

The following steps provide a rough guideline to performing EVT without the use of a specialized kit (eg, Endo-SPONGE or Eso-SPONGE; B. Braun, Melsungen, Germany), as is commonly performed in the United States. Table 1 lists the typical equipment used during EVT. Acknowledging that EVT can be performed with a variety of techniques and methods, the following steps simply represent 1 method of performing EVT.

1. *Endoscopic examination of the defect.* On identifying the defect(s), a thorough examination of the defect is performed to ascertain the dimensions of the defect, the location of the defect, and the character of the cavity. This aids in determining the size of the intended sponge and whether the sponge will be placed inside the cavity (intracavitary) or in the adjacent lumen (intraluminal). Typically, intracavitary placement is preferred because it maximizes the formation of granulation tissue by increasing contact between the sponge and the tissue within the defect. However, in smaller defects (<1 cm diameter), intracavitary placement of the sponge is not possible, and intraluminal placement is performed to fully cover the defect. Endoscopic examination can also be performed with or without fluoroscopy depending on endoscopist preference. Vigorous water irrigation and suctioning with removal of any debris or food material is also performed to clean the cavity before sponge placement.
2. *NGT (for upper GI tract) insertion.* A 12F to 18F NGT is advanced through the naris into the posterior pharynx. The tip of the NGT can then be grasped either with one’s fingers or using an endoscope with a grasping forceps. The tip is then brought out of the mouth where a sponge can be affixed to it. Alternatively, instead of retrieving the NGT from the posterior pharynx, the procedure can be performed with the NGT exiting through the patient’s mouth. The NGT can then be transferred from the mouth to the patient’s naris using a transfer device (similar to a nasal transfer when placing a nasobiliary drain).
3. *Customize the sponge.* The sponge is composed of polyurethane foam. Sponges found in postoperative wound vacuum kits can generally be used. Based on the endoscopic evaluation, a sponge is cut (using scissors) into the dimensions (Video 1, available online at www.igiejournal.org) that would fit into the cavity (intracavitary) or the adjacent lumen (intraluminal). The maximum sponge (noncompressed) diameter will be limited to the diameter of the upper esophageal sphincter in the upper GI tract and the diameter of the anus in the lower GI tract because the sponge will need to be driven past these orifices. The minimum sponge size is dictated by the size of the NGT.
4. *Fix the sponge to the NGT.* After creating the appropriate size sponge, the sponge is sutured to the NGT. A standard needle driver can be used to create a hole through the vertical axis of the sponge followed by grasping the distal tip of the NGT to pull it through such that the sponge covers the fenestrated portion of the NGT and the NGT is coaxially located through the sponge (Video 2, available online at www.igiejournal.org). Permanent sutures are then placed through the sponge and the NGT to secure the sponge to the tube in at least 2 locations (distal and proximal end of the sponge) with typically a third suture placed in the center of the sponge. The final suture is placed through the tube and sponge at the distal end of the tube with creation of a loop that can be grasped endoscopically.
5. *Endoscopic placement of the sponge.* A grasping forceps is passed down the working channel of the endoscope and used to grab the looped suture at the distal end of the NGT with the affixed sponge. The endoscope and the affixed sponge are then advanced in parallel through the mouth or the rectum (Video 3, available online at www.igiejournal.org). Advancement of the endoscope with the affixed sponge through the oropharynx and upper esophageal sphincter is often met with significant resistance; thus, generous amounts of lubricant should be applied to both the endoscope and sponge before insertion. The endoscope with affixed sponge is gradually advanced to the site of the defect. Precise placement of the sponge can be performed using the grasping forceps while the endoscope is slowly withdrawn. Once the sponge is in the proper position, the endoscopist releases the suture loop from the grasping forceps. Because of the significant amount of tension, releasing the suture from the forceps may prove difficult, requiring the endoscopist to move the forceps back and forth and side to side in quick movements (jiggling maneuver) until the forceps releases. The endoscope is then withdrawn from the patient, leaving the NGT with sponge in position. In challenging cases of attempted intracavitary placement, an alternative technique entails the use of an overtube where the endoscope and overtube are advanced into the cavity.^10^ With or without fluoroscopy, the endoscope is then removed with the overtube left in place. The NGT with the affixed sponge is then advanced through the overtube into the cavity. If fluoroscopy is not being used, the endoscope can be reinserted through the overtube to confirm correct placement. The end of the NGT coming out of the orifice must then be cut to allow for removal of the overtube while keeping the sponge in place. Note that with this technique, nasal transfer is performed after sponge insertion using a transfer device (as described in step 2).
6. *Tube fixation.* In the upper GI tract, once the affixed sponge is in place, the NGT is secured to the nose using a nasal bridle, suturing, or taping the tube to the nose. Once secure, the NGT is connected to suction. For lower GI tract EVT, fixation is typically not needed with the suction effect alone sufficient for keeping the sponge in place. When the patient is traveling, suction can be administered with a portable suction canister, and when stationary, wall suction can be used (if available). Negative pressure is typically administered between 100 and 175 mm Hg (125 mm Hg is most common). The rationale of this pressure range stems from initial studies in animals and humans where the application of 125 mm Hg of pressure for wound vacuum therapy to skin defects led to the increased local blood flow and faster granulation tissue formation.^1^^,^^11^ Retrospective series from a single center have demonstrated that lower pressure (50 mm Hg) EVT is effective in esophageal defects without any major bleeding events found, but further investigation is needed to identify the optimal negative pressure for EVT within the GI tract.^12^^,^^13^
7. *Exchanges.* Replacement of the sponge is performed every 3 to 5 days until full healing occurs (usually 2-3 weeks). Replacements are required to prevent tissue ingrowth into the sponge and enable modification of the sponge size and placement location in response to healing. With the suction turned off, a grasping forceps is used to grasp the NGT proximal to the sponge, and the sponge is removed through the mouth or rectum. Endoscopic examination of the defect is then performed, followed by placement of a new customized sponge. Sponge exchanges are generally performed with the patient under general anesthesia with endotracheal intubation given the uncomfortable nature of the exchange; however, exchange procedures can be performed with the patient under deep sedation at the discretion of the endoscopist and treatment institution. Patients are typically kept in the hospital until final removal of the sponge given the need for frequent exchanges.
8. *Postprocedure care.* Between exchanges, supportive care typically entails administration of antibiotics and nutrition. For upper GI defects, nutritional support includes either total parenteral nutrition or tube feeds because oral nutrition should not be administered. Total parenteral nutrition carries an advantage over tube feeds in that it does not need to be held for exchange procedures; however, it always carries the risk of infection. If tube feeds are given, jejunal feeding is preferred through the jejunostomy tube or an extension gastrojejunostomy tube (in patients who already have a gastrostomy tube). In lower GI defects, oral nutrition can be given if there is a diverting stoma proximal to the defect because stool contents can result in migration of the sponge or occlude the sponge. In the absence of a diverting stoma, total parenteral nutrition is the preferable option.
9. *Assessment of healing.* EVT may be considered successful when the leak or defect has completely closed and continuity of the GI tract has been re-established. The sponge is removed at this point, and a contrast study (ie, upper GI series or lower GI series) can be performed to confirm closure of the defect. At a certain point during EVT, maximum progress may be seen with no further improvement found during sponge exchanges. EVT may be stopped at this point with primary closure attempted endoscopically or surgically. Additionally, during the course of EVT, a patient demonstrating clinical deterioration may indicate that either the sponge has migrated or efficacy with EVT is lacking. Depending on the stability of the patient, endoscopic assessment is warranted to identify the cause of clinical worsening.
10. *Follow-up after healing.* Once complete healing has been achieved, patients may resume an oral diet. Endoscopic follow-up is not inherently necessary, although if the patient develops dysphagia (in upper GI cases), endoscopic evaluation can help identify the formation of a stricture.

**Table 1:** Examples of equipment used to perform endoscopic vacuum therapy

Manufacturer	Item	Description
Standard endoscopic vacuum therapy		
3M (St Paul, Minn, USA)	V.A.C Granufoam dressing	Sponge material
Covidien (Mansfield, Mass, USA)	Nasogastric tube (12-18F)	Drainage tube
Ethicon (Somerville, NJ, USA)	Strong permanent suture	Suture used to fix the sponge to the drainage tube
Steris (Mentor, Ohio, USA)	Raptor grasping device	Forceps used to grasp the distal suture or the drainage tube
Olympus (Center Valley, Pa, USA)	Rat-tooth alligator jaw grasping forceps	Forceps used to grasp the distal suture or the drainage tube
AMT (Brecksville, Ohio, USA)	Nasal bridle (upper GI tract)	Attachment device to secure drainage tube to nose
Hollister (Libertyville, Ohio, USA)	Feeding tube attachment (upper GI tract)	Nasal tape to hold drainage tube to nose
Modified endoscopic vacuum therapy		
3M	Ioban 2 Antimicrobial Incise Drape	Antimicrobial drape used to cover gauze and drainage tube for modified endoscopic vacuum therapy

### Premade EVT products

Of note, several proprietary EVT products are not currently available in the United States but are available globally. The Eso-SPONGE system (Braun) is designed for use in the upper GI tract and comes with an open-pored polyurethane sponge prefitted to a drainage tube. When using this device, the endoscope is used to identify the defect, allowing subsequent placement of an overtube leading to the defect. Once the sponge is modified to the desired size, the sponge is advanced through the overtube into the cavity. The overtube can then be removed, and an oral–nasal transfer is performed.

**Figure 1:**
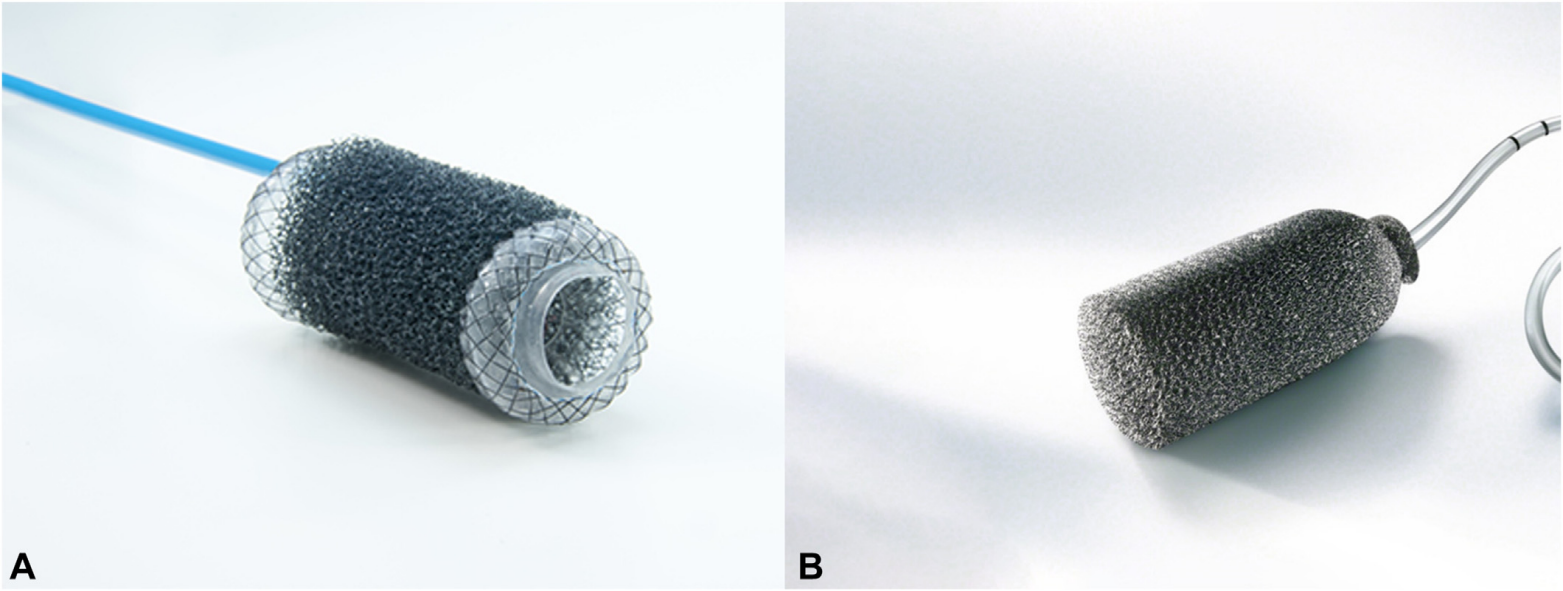
Example of a ready-made endoscopic vacuum therapy kits. **A,** A polyurethane sponge overlying a fully covered metal stent (VacStent GI; VAC Stent GmbH, Fulda, Germany). **B,** Premade polyurethane sponge (Endo-SPONGE; B. Braun, Melsungen, Germany) connected to a drainage tube. (Images provided courtesy of Möller Medical and Braun.)

The Endo-SPONGE (Braun) is designed for use in the lower GI tract and, similar to the Eso-SPONGE system, comes with a prefitted polyurethane sponge connected to a drainage tube (Fig. 1A). The endoscope and overtube are advanced to the defect. The overtube is left in place, and the endoscope is removed. Once modified to the appropriate size, the lubricated sponge is advanced through the overtube into the defect. The overtube is then removed, and the drainage tube is connected to a low-vacuum bottle, included in the kit.

A more recently developed system (VacStent GI; VAC Stent GmbH, Fulda, Germany) combines a fully covered metal stent (nitinol stent with a silicone-membrane covering) with an overlying polyurethane sponge cylinder (Fig. 1B) for the treatment of upper GI leaks.^14^^,^^15^ Under fluoroscopy, the introducer (12-mm diameter) is advanced through the mouth over a guidewire to the desired location, thereby allowing stent deployment. The stent is 30 mm in diameter at its flanges. The sponge, which covers the central (nonflanged ends) portion of the stent, is 50 mm in length and 10 mm thick. The sponge comes connected to a 12F catheter that is affixed to an external vacuum pump once repositioned transnasally.

### Modified EVT techniques

A number of alternative techniques for performing EVT have been described. The most well-described technique entails the use of a film to serve as a surrogate for the sponge. This open-pore drainage film (Suprasorb CNP Drainage Film; Lohmann & Rauscher International, Rengsdorf, Germany) is wrapped around the distal end of an NGT and sutured to the tube.^16^ In this manner, the drainage tube has a diameter of only 4 to 6 mm and can be inserted directly through the nose, as opposed to the mouth with the traditional EVT technique. A suture loop is attached to the most distal portion of the NGT to allow for maneuverability with grasping forceps during endoscopy. If a double-lumen or triple-lumen tube with a jejunal feeding tube extension is used, enteral nutrition can also be administered through the tube while also applying negative pressure. This technique has been successful in treating a number of defects, including upper and lower GI anastomotic leaks, duodenal perforations, and infected pancreatic necrosis.^16^, ^17^, ^18^, ^19^ The primary benefit of this technique is the small size of the tube, which can be easier to place into smaller cavities because of increased maneuverability through tight spaces (eg, upper esophageal sphincter).

**Figure 2:**
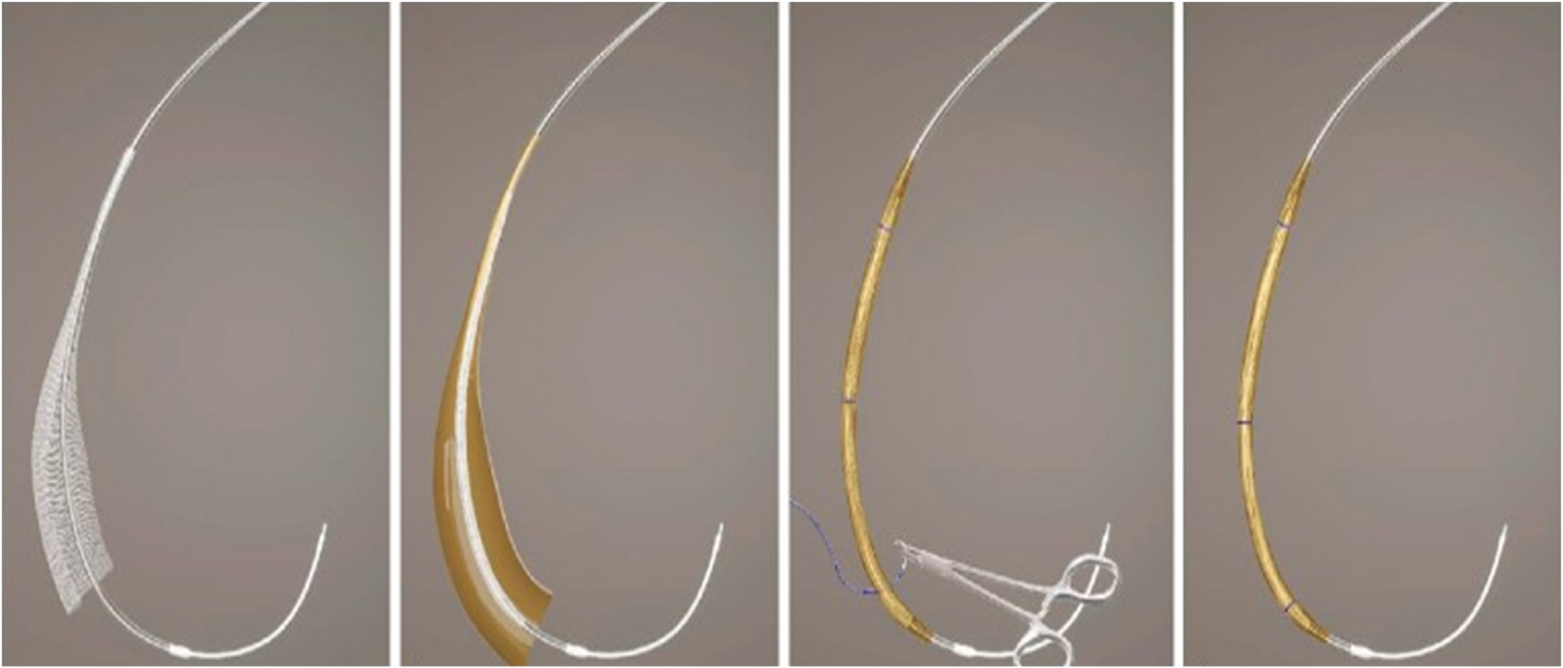
Example of modified endoscopic vacuum therapy techniques including the use of an antimicrobial drape wrapped around a nasogastric tube. (*Panel 1, leftmost*) Gauze being wrapped around a nasogastric tube. (*Panel 2*) Antimicrobial wrap being placed around the gauze. (*Panel 3*) Suturing of antimicrobial wrap and gauze to the nasogastric tube. (*Panel 4, rightmost*) Modified sponge utilizing antimicrobial wrap and gauze. (With permission from De Moura D, et al. Endoscopy 2022;54:E837-9.)

Because the open-pore drainage film is costly and currently unavailable in the United States, a cost-effective modification of this technique has been developed using gauze and an antimicrobial drape in lieu of the drainage film (Fig. 2).^20^ Briefly, a sheet of gauze is cut to cover only the fenestrated portion of an NGT. The gauze is wrapped around all fenestrations. The antimicrobial drape is then cut to match the size of the gauze and is wrapped around the gauze. The gauze and drape thus serve as the “sponge,” subsequently secured by suturing to the NGT. An 18-gauge needle is then used to puncture through the sponge and tube to create additional drainage holes. In a retrospective multicenter study, this technique was used in 144 patients with upper and lower GI tract defects with a clinical success rate of 88.9%.^21^

## Clinical Uses of EVT

### Postesophagectomy anastomotic leaks and fistulae

Although esophagectomy remains the treatment of choice for locally advanced esophageal cancers, anastomotic leaks represent a relatively common adverse event with associated mortality rates as high as 35%.^22^ Although placement of a covered metal stent has become the endoscopic treatment of choice for such leaks, EVT offers an alternative treatment option.^23^

A meta-analysis reporting on EVT for anastomotic leaks included 14 studies with 248 patients and demonstrated a closure rate of 79.5%.^24^ In comparing esophageal stent placement with EVT, the same meta-analysis revealed that closure with EVT was superior to stent placement (risk difference, .26). A similar meta-analysis on anastomotic leaks including 7 observational studies (338 patients) directly comparing EVT with stent placement found that EVT had a significantly higher closure rate (odds ratio, 2.47; 95% confidence interval, 1.30-4.90), along with a more favorable treatment duration (median difference, 11.57 days).^25^ A recent multicenter prospective study examining a premade EVT kit (Eso-SPONGE; Braun) for the treatment of anastomotic leaks in 69 patients revealed a closure rate of 91.3% with a mean treatment duration of 25 days.^26^ Of note, no randomized trials to date have directly compared stent therapy with EVT for the treatment of anastomotic leaks.

Fistulae represent progression from a prolonged anastomotic leak to an epithelialized connection that develops between the esophagus and adjacent organs or cavities. Although challenging to manage because of their fibrotic nature and inflammatory surrounding tissue, EVT provides another treatment option. In limited case series, successful fistula closure was found in approximately 50% of cases treated with EVT.^27^^,^^28^ Of note, EVT should generally be avoided for the management of tracheoesophageal fistulae given the danger of applying negative pressure within the airway. Although EVT has been reported as a successful salvage treatment for small bronchoesophageal fistulae and small tracheoesophageal fistulae close to the tracheal bifurcation, it is critical to recognize that fistula formation represents a potential adverse event of EVT and caution is warranted whenever applying negative pressure to the airway.^27^^,^^29^

### Upper GI perforations

In managing defects not related to surgery (eg, spontaneous, endoscopy-related, and foreign-body perforations) within the upper GI tract, EVT offers an alternative treatment to surgical repair or other endoscopic modalities such as stent placement, endoscopic suturing, or clip closure, particularly for defects too large for standard endoscopic techniques (Video 4, available online at www.igiejournal.org). For esophageal perforations, a retrospective study of patients with iatrogenic, spontaneous, and foreign body–related perforations found successful closure with EVT in 9 of 10 patients after a mean treatment duration of 19 days. Similarly, small studies examining iatrogenic (endoscopy-related) perforations found 100% closure rates with the use of EVT.^30^^,^^31^ In a recent multicenter retrospective cohort study, EVT was used as the initial therapy for acute esophageal perforations in 27 patients (59% iatrogenic, 33% Boerhaave syndrome) with successful healing observed in 89% of patients.^32^ EVT was initiated in this study after a median of 1 day after perforation with a mean treatment duration of 12 days involving 1 sponge exchange on average. In a prospective multicenter study, EVT with a proprietary system (Eso-SPONGE; Braun) was performed in 33 patients with spontaneous and iatrogenic esophageal perforations, with a mean perforation diameter of 2 cm and depth of 4.8 cm.^26^ The perforation successfully closed using EVT in 75.8% of patients after a mean treatment duration of 30 days.

EVT has also been reported as a treatment option for duodenal perforations. In a 2-center retrospective study, EVT led to definitive closure in 80% of patients (8/10) with duodenal perforations after a mean treatment duration of 12 days.^33^ EVT was used as the first-line therapy in 7 patients and as the second-line treatment in 3 patients. In addition to numerous case reports describing successful use of EVT for duodenal perforations (after failure with stent placement), EVT has also been used as the first-line therapy for spontaneous duodenal diverticula perforations, leading to successful nonsurgical resolution.^34^ Caution should be given to avoid placing the sponge near the ampulla because of the risk of biliary or pancreatic obstruction.

### Leaks occurring after bariatric surgery

With over 250,000 bariatric surgical procedures performed in the United States in 2021, this subgroup of patients is at risk for gastric defects occurring after bariatric surgery.^35^ Staple line defects can occur in 1% to 2% of patients after sleeve gastrectomy and in 2% to 5% of patients after Roux-en-Y gastric bypass.^36^^,^^37^ Although true ischemic leaks are possible, most postbariatric leaks occur because of increased intragastric pressure exceeding the staple line resistance, especially after sleeve gastrectomy, and are located at the proximal end of the staple line. Because symptom onset is insidious, with almost 50% of patients asymptomatic because of visceral fat restricting the leak and preventing peritonitis, and revisional surgery is limited beyond the second postoperative day, management typically consists of endoscopic measures, including through-the-scope-clip closure, over-the-scope-clip closure, endoscopic suturing, endoscopic luminal stent placement, and double-pigtail plastic stent placement into the cavity. Data on the utility of EVT in management of postbariatric leaks are encouraging, with a recent systematic review and meta-analysis of 5 studies with 55 patients yielding a high clinical success rate (87.2%), with a modest (6%) adverse event rate and a 12.5% rate of dislodgements requiring sponge exchange.^38^

### Colorectal anastomotic leaks

Postsurgical colorectal anastomotic dehiscence and subsequent leaks occur in 2% to 7% of patients, with a higher incidence in coloanal than ileocolic anastomoses, carrying a significantly increased risk of sepsis and chronic fistula formation with a mortality rate of up to 12%.^39^^,^^40^ Management varies depending on several factors (eg, location, infection, overall comorbidities, etc), and surgery is typically indicated for those with sepsis and signs of peritonitis. Unfortunately, surgical repair often results in discontinuity of the GI tract. For hemodynamically stable patients, endoscopic therapy can be considered, with options including over-the-scope clip closure and metal stent placement for smaller leaks and EVT reserved for larger defects (>2 cm) associated with a cavity and/or abscess.

A meta-analysis of 17 studies with 384 patients who had anastomotic leaks reported a very high technical success rate (99.9%), a 84.9% pooled clinical success rate, and a pooled adverse event rate of 7.6%, with recurrent abscess formation (.5%) and bleeding (.4%) as the most common adverse events.^41^ Leaks were detected after a mean of 28 days (range, 7.1-85), and the mean duration of EVT was 33 days (range, 15-108), including a mean of 8.2 procedures (range, 2.2-16.2). The mean follow-up time was 18.2 months (range, 2-48). This study also demonstrated improved clinical success if EVT was used as an early intervention rather than late intervention (beyond 15 days). Furthermore, neoadjuvant chemoradiation therapy affected EVT duration (requiring longer treatment) and success (requiring higher number of sponge exchanges). Another systematic review and meta-analysis including 24 studies and 690 patients revealed a similarly high clinical success rate (81.4%).^42^ This study, however, found a 12.1% pooled adverse event rate with EVT including anastomotic stenosis (0%-18.2%), fistula formation (0%-28.6%), abscess and chronic sinus persisting >12 months (0%-21.7%), and bleeding (0%-9.7%). Bowel diversion may still be necessary for effectiveness when colorectal leaks are treated with EVT. In this meta-analysis, the weighted mean fecal diversion rate was 76%; however, eventual ostomy reversal was feasible in 63% of cases. These data support EVT as a viable and effective minimally invasive treatment option for colorectal leaks.

### Prophylactic EVT

EVT has been used prophylactically in patients undergoing major esophageal surgery with high-risk anastomoses, to treat small undetectable anastomotic defects, and to prevent leaks with mediastinal contamination. A small number of studies have described intraoperative placement of EVT intraluminally at the anastomosis with re-evaluation 3 to 6 days later, reporting anastomotic leak rates between 0% and 7.5%, which is lower than reported leak rates (10%-20%) with conventional esophageal surgery principles.^43^^,^^44^ Available data suggest extending EVT beyond 3 to 6 days if any high-risk features are seen, including visible suture material and fibrin and ischemic changes, thereby making preemptive EVT an attractive option. A recent systematic analysis detailing the available literature (5 case reports, 5 retrospective case series, and 1 retrospective case-control study) reported no major EVT-related adverse events and no mortality attributable to this intervention. More data in the form of randomized trials are needed to prove its safety and efficacy in a prophylactic role.^45^

## Adverse Events

The primary major adverse events of EVT include stricture formation, bleeding, and fistula formation.^46^ If bleeding occurs, EVT should be discontinued given the high risk of significant bleeding with persistent negative pressure. Stricture formation after EVT treatment can be treated endoscopically, whereas fistula formation may require surgical intervention.

## Pros and Cons

EVT adds to the existing armamentarium of endoscopic options for the management of luminal defects by using devices and instruments widely available at most medical centers. This method facilitates drainage of the cavity (reducing the need for secondary external drainage) while simultaneously providing an environment for wound healing. At the same time, the disadvantages of EVT include the need for multiple sequential endoscopic procedures (typically in the inpatient setting), patient discomfort because of transnasal tube placement, and reduced oral intake because of intentional occlusion of the GI tract.

It is noteworthy that there are several alternative endoscopic techniques for management of leaks and perforations, with endoscopic clips and suturing showing high success rates in acute perforations with small defects and no associated cavity.^47^^,^^48^ Self-expandable metal stents are the most common alternatives and remain the mainstay of treatment at many centers, with overall clinical success rates of 80% to 90%. The main limitation of self-expandable metal stents remains the lack of cavity drainage external to the stent.^23^^,^^49^ Available observational data comparing the outcomes between self-expandable metal stents and EVT favor EVT with higher success rates, shorter duration of therapy, and lower adverse events, although an ongoing randomized trial may provide further clarity.^50^, ^51^, ^52^, ^53^

## Financial Considerations

There is no designated Current Procedural Terminology code for EVT. Therefore, when billing EVT, Current Procedural Terminology codes 43999 (stomach), 43499 (esophagus), or 45999 (colon and rectum) are typically used depending on the site of treatment. Fee calculation can be compared with performing an upper endoscopy (43235) or colonoscopy (45378) and negative pressure wound therapy (97605). A modifier 22 can be incorporated to the billing to reflect the relative complexity of performing EVT in comparison with a standard endoscopy.

Patients are kept in the inpatient setting for the duration of therapy and, as mentioned previously, with repeat endoscopic procedures every 3 to 5 days. It bears mentioning that the long hospitalizations and frequent procedures associated with EVT pose a significant cost to both patients and the healthcare system. This cost and morbidity, however, needs to be compared with the alternative, which could include an operative and/or nonoperative intervention-related prolonged hospital course, additional adverse events, and severe disability and/or death related to these adverse events. It is well recognized that these patients are among the most challenging to manage from a clinical and technical perspective, and EVT represents an innovative, minimally invasive approach that has helped avoid more costly surgical reinterventions and additional morbidity based on the data so far.

## Summary

Drawing on the principles of vacuum-assisted closure of wounds in the skin, EVT represents an effective salvage endoscopic approach to the treatment of defects throughout the GI tract. By using negative pressure to drain infected fluid and remove debris, EVT provides source control while also promoting cavity collapse and the development of granulation tissue for healing. Although proprietary EVT kits are not currently available in the United States, EVT can be performed in the endoscopy unit with readily available materials. Given the intensive nature of therapy with repeat endoscopies every 3 to 5 days for several weeks, current barriers include patient tolerance, endoscopist training and comfort (likely requires several trained endoscopists at each center given need for frequent exchanges), resource availability, and the likely financial loss with performing this treatment. Randomized studies are needed to compare EVT with other treatment modalities, and continued innovation is required for widespread adoption in the future. These data will be important for appropriate positioning of this treatment in the management algorithm but also very helpful in obtaining a Current Procedural Terminology code for this intervention with an aim toward reimbursement for this procedure.

## Disclosure

The following authors disclosed financial relationships: V. S. Akshintala: Board member for Origin Endoscopy Inc; consultant for Dragonfly Endoscopy Inc; research support from Abbvie, Boston Scientific Corporation, and Medtronic; travel compensation from Mauna Kea Technologies, Inc; food and beverage compensation from Mauna Kea Technologies, Inc, Boston Scientific Corporation, and AbbVie Inc. D. Chen: Food and beverage compensation from Ambu Inc. Y. Chen: President of Chess Medical; consultant for Boston Scientific Corporation. K. K. Das: Patent with Interpace Biosciences; food and beverage compensation from Medtronic, Inc. G. Leung: Consultant for Boston Scientific Corporation and Steris Corporation. G. Mishra: Consultant for Pentax of America, Inc and Cook Medical LLC; travel compensation from Pentax of America, Inc; food and beverage compensation from Pentax of America, Inc, Cook Medical LLC, Ambu Inc, Boston Scientific Corporation, and Wilson Cook Medical Incorporated. V. Raman Muthusamy: Consultant for Medtronic and Boston Scientific Corporation; research support from Boston Scientific Corporation; stock options/equity in Capsovision; advisory board for Endogastric Solutions and Motus GI; travel compensation from Boston Scientific Corporation; food and beverage compensation from Boston Scientific Corporation, Steris Corporation, Medtronic, Inc, Apollo Endosurgery US Inc, Endogastric Solutions, Inc, Cook Medical LLC, Pentax of America, Inc, and Olympus America Inc. F. U. Onyibma: Food and beverage compensation from Endogastric Solutions, Inc and Medtronic, Inc. S. Pawa: Consultant for Boston Scientific Corporation. T. Rustagi: Consultant for Boston Scientific Corporation; food and beverage compensation from Boston Scientific Corporation and Merit Medical Systems Inc. S. Sakaria: Food and beverage compensation from AbbVie Inc. R. J. Law: Consultant for Conmed Corporation, Boston Scientific Corporation, Medtronic USA Inc, and Olympus America Inc; royalties from UpToDate; food and beverage compensation from Olympus America Inc, Boston Scientific Corporation, and Ethicon Inc. All other authors disclosed no financial relationships.

## Supplementary data

Video 1 Customization of sponge to size of defect.

Video 2 Fixation of sponge to nasogastric tube.

Video 3 Advanced of sponge and nasogastric tube to the defect.

Video 4 Example of healing of esophageal perforation treated with endoscopic vacuum therapy.
